# Landscape of Gene Essentiality in Cancer Cell Death Pathways

**DOI:** 10.3390/genes17040491

**Published:** 2026-04-21

**Authors:** Shangjia Li, Zhimo Zhu, Chen Yang, Nuo Sun, Lijun Cheng, Lang Li

**Affiliations:** 1 Department of Biomedical Informatics, College of Medicine, The Ohio State University, Columbus, OH 43210, USA; 2 Department of Physiology and Cell Biology, College of Medicine, The Ohio State University, Columbus, OH 43210, USA

**Keywords:** regulated cell death, cancer, gene essentiality

## Abstract

Background/Objectives: Regulated cell death (RCD), a process that relies on a series of molecular mechanisms, can be targeted to eliminate superfluous, irreversibly damaged, and potentially harmful cells. In this research, we want to better understand how the cell death pathway contributes to cancer therapy. Methods: We studied 1150 cancer cells in the Dependency Map (DepMap) database for 12 distinct cell death pathways and assessed their gene essentialities. Genes which are essential in 90% or more of cancer cell lines are called always essential, or partial essential if falling into (10%, 90%), or rare essential if they are essential in less than 10% of cancer cell lines. Results: Overall, among these 12 cell death pathways, 23, 47, and 549 genes were classified as always essential, partial essential, and rare essential, respectively. In two cell death pathways, Parthanatos, and Pyroptosis, all genes were rare essential. Among the other ten cell death pathways, Apoptosis, Autosis, Necroptosis, Efferocytosis, Ferroptosis, Mitotic cell death, Autophagy, Lysosome-dependent cell death, MPT-driven necrosis and Immunogenic, there are (10, 1, 13, 6, 3, 9, 11, 1, 1, 0) partial essential genes, and (2, 0, 3, 1, 1, 13, 4, 0, 0, 1) always essential genes. Conclusions: These cell death pathway essential genes could be viable targets for therapeutic drug development for cancer therapies.

## 1. Introduction

The regulated cell death was first observed by Karl vogt in 1842. The definition and description of the regulated cell death pathway appeared relatively late. Apoptosis was described by Kerr et al. in 1972, in which Apoptosis was defined as a cell morphological change including the nucleus comprising and cytoplasmic condensation and breaking up [1]. And in 1980, Wyllie et al. described the characteristics and the importance of Apoptosis [2]. Autophagy was coined by de Duve in 1963. It was defined as the cellular process through which intracellular materials are delivered to the Lysosome for degradation [3]. Since then, a great deal of experimental evidence has accumulated, in both molecular and morphological levels, in studying regulated cell death. In the paper published by the Nomenclature Committee on Cell Death (NCCD) in 2018 [4], twelve cell death pathways were summarized, including Apoptosis, Autosis, Autophagy, Necroptosis, Efferocytosis, Ferroptosis, Immunogenic cell death, Lysosomal cell death, Mitotic cell death, Mitochondria Permeability Transition cell death, Parthanatos and Pyroptosis.

Apoptosis can be separated into intrinsic Apoptosis and extrinsic Apoptosis, through their different upstream signaling pathway. The extrinsic Apoptosis is activated by the cell death receptors, such Fas and TNFR1, located on the cell membrane, while the intrinsic Apoptosis is triggered by the permeabilization of the mitochondrial outer membrane which is induced by the BCL2 family proteins [5]. Autophagy is a process of self-degradation of cellular components. It starts from the formation of autophagosome, and later on, fuses with Lysosomes for cargo deration. During this Autophagy process, mTORC1 complex, ULK complex and PI3K complex all play critical roles [6]. Necroptosis is another well-studied regulated cell death pathway that can be initiated by various stimuli, including TRAIL, TNFR1, and Fas, among others. Necroptosis leads to Caspase-independent cell “suicide” and involves multiple processes such as the generation of reactive oxygen species (ROS), calcium overload, and chromatin lysis.

Efferocytosis is a process to clean up the apoptotic cells. It is regulated by three distinctive types of signaling: (1) “Find me” signaling controls the phagocyte recruitment; (2) “Eat me” signaling controls the process of recognition and engulfment; and “Don’t eat me” signaling inhibits the phagosome phagocytosis. Efferocytosis typically undergoes the fusion of apoptotic fusion with endosome and the endosome fusion with Lysosome [7]. Autosis is a form of autophagic cell death with unique morphological features. It is triggered by the Na^+^/K^+^-ATPase interaction with Becline1 [8]. Ferroptosis is an iron-dependent cell death pathway, which is executed by accumulated iron and the production of ROS [4]. Mitotic cell death eliminates the cells that are unable to complete the Mitotic. Although the molecular mechanism of Mitotic cell death is not fully characterized, it has been shown that the DNA damage and inappropriate activation of the CDK1/cyclin B1 complex could induce the Mitotic cell death. Lysosomal cell death goes through the Lysosomal membrane permeabilization. It releases cathepsin which leads the activation of CCCaspase-3 (Caspase-dependent cell death) and AIF (Caspase-independent cell death) [9,10].

Mitochondria Permeability Transition (MPT) cell death is induced by oxidative stress and cytosolic Ca^2+^ overload. MPT is regulated by the PTPC protein complex. It leads to mitochondrial permeability transition, and then triggers Apoptosis or Necroptosis [11]. Pyroptosis is initiated by the assembly of inflammasome. The assembled inflammasome induces the Caspase-1, and subsequently, Caspase-1 leads to the execution protein activation and pore formation on the cell membrane, and eventually cell death [12]. Parthanatos is a cell death pathway triggered by Ca^2+^ overload and DNA damage. DNA damage leads to the overactivation of PARP1, which causes AIF to translocate into the nucleus and fragment DNA [13]. Immunogenic cell death pathway is a form of cell death which can identify dying cells and is a major trigger of adaptive immunity [14].

Cell death pathways are generally complex biological processes. In cell death regulation, it is not clear which genes are more important than others, and whether there are variations among cancer cells. Clustered regularly interspaced short palindromic repeats (CRISPR) Cas9 system is a powerful gene knockout technology. It allows us to investigate cell death due to individual gene knockout, i.e., gene essentiality. Using genome-wide CRISPR/Cas9 technology, the Dependency Map (DepMap) project screened 18,000 gene essentialities across more than 1100 cancer cell lines [15]. In this paper, we will use the DepMap data to identify essential genes among 12 cell death pathways and evaluate their essentiality among these cancer cells.

## 2. Materials and Methods

All analyses were implemented in Python (version 3.11.4) using custom scripts. Data preprocessing and matrix organization were performed using pandas (version 2.3) [16] and numpy (version 2.2.0) [17], and data visualization was conducted using matplotlib (3.10.8) [18] and seaborn (version v0.13.2) [19].

### 2.1. Cell Death Pathway Data Collection

Cell death-associated genes were compiled and integrated from multiple resources, including the literature review from PubMed and web of science and google scholar, KEGG [20] and XDeathDB [21]. The selection of cell death pathways was primarily based on XDeathDB and the guidelines published by the Cell Death Nomenclature Committee in 2018 [4]. When constructing cell death pathways, Apoptosis, Autophagy, Necroptosis, Efferocytosis, and Ferroptosis were collected from the KEGG database [20]. The other pathways, including Autosis [8,22,23,24,25,26,27,28,29], Mitotic cell death [9,10,30,31,32,33,34,35,36,37,38,39], Lysosome-dependent cell death [40,41,42,43,44,45,46,47], MPT-driven necrosis [4,48,49,50,51,52,53,54,55,56,57,58,59,60,61,62,63,64,65,66], Immunogenic cell death [67,68,69,70,71,72,73,74,75,76,77], Parthanatos [13,78,79,80,81,82,83,84,85,86,87,88,89,90,91], and Pyroptosis [92,93,94,95,96,97,98,99,100,101,102,103,104,105,106,107,108,109] were built upon an extensive literature review. To construct the cell death pathway:

1. Start with a list of genes from the XDeath database and the paper published by the Cell Death Committee in 2018.
2. Search for statements using the terms: “[Name of cell death pathway]” and “Cell death pathway”. Pathways from multiple papers will be combined, keeping overlapping parts and searching for materials to address non-overlapping sections. This step will help identify the functions and positions of the genes in the pathway and may also reveal new genes to include.
3. For genes not mentioned in the literature, search using “[Gene Name],” “[Name of cell death pathway],” and “Cell death” or using another group of term: “[Upstream gene]” and “[Downstream gene]” and “interaction” and “Cell death” to clarify their roles.

### 2.2. DepMap Data Collection

The gene essentiality data were obtained from the Dependency Map (DepMap) portal (https://depmap.org/portal/) (accessed on 11 November 2024). Specifically, the dataset is available via the following link: https://plus.figshare.com/articles/dataset/DepMap_24Q2_Public/25880521/1 (accessed on 11 November 2024). This dataset provides a gene essentiality score for 18,443 genes among 1150 cancer cell lines [12].

### 2.3. Gene Essentiality Classification

In the DepMap database, the gene fitness effect score is defined as the relative change in growth rate before and after a gene is knocked out in a specific cell line. These scores are inferred using the Chronos algorithm [110]. The read counts, derived from genome-wide and large sub-genome loss-of-function CRISPR screens, are processed through the Chronos workflow. Chronos is an algorithm that can calculate the fitness effect of a gene to the cell in CRISPR screens. It can determine the effect of gene knockout on cell growth rate using the sgRNA deletions across screens and time points. The resulting inferred gene fitness effects are subsequently corrected for copy number variations to ensure accuracy. The essential genes are defined from gene fitness effect score [110]. A cutoff value of −1 was used. Specifically, essential genes are genes whose gene fitness effect score are below −1. Otherwise, they are non-essential.

Using this criterion, we further categorized genes based on their essentiality frequency across all cancer cell lines:

- Always essential genes (AEGs): genes classified as essential in more than 90% of cell lines.
- Rare essential genes (REGs): genes classified as essential in fewer than 10% of cell lines.
- Partially essential genes (PEGs): genes classified as essential between 10% and 90% of cell lines.

In each cell death pathway, we calculated the proportion of AEGs, REGs, and PEGs.

### 2.4. Clustering Analysis

Clustered heatmaps were generated in Python (version 3.11.4) using seaborn.clustermap() (version v0.13.2) on the gene essentiality matrix, where rows represented genes and columns represented cell lines. Each cell contained the corresponding gene essentiality score and was used to visualize gene essentiality patterns and similarities among genes and cell lines [19]. Gene fitness effect scores were visualized as a heatmap, with hierarchical clustering applied to both genes and cell lines.

### 2.5. Gene Publication Trends Analysis

We downloaded all abstracts related to cancer from Pubmed as a corpus. To classify the experimental maturity of abstracts (e.g., in vitro, in vivo, or ex vivo), we first employed BioBERT as a diagnostic stress test for our annotation guidelines. Unlike Large Language Models (LLMs) that can “guess” intent, BioBERT’s more literal processing highlighted exactly where the human-designed rules were ambiguous or inconsistent [111]. By identifying where the model plateaued, we were able to refine the annotation schema to be more scientifically rigorous before scaling the analysis. We then leveraged in-context learning using MedGemma-27b and refined schema to classify the corpus [112,113]. A final BioBERT model was distilled from MedGemma-27b to perform large-scale, cost-effective inference across the entire corpus.

To determine how often specific genes were studied, we performed gene entity normalization using HGNC symbols and aliases. These candidates were then verified using MedGemma-27B as a teacher model to filter out ambiguous mentions and confirm the gene was the true subject of the research.

We used the model to quantify gene-specific publication frequency. As this model is part of an ongoing study in our laboratory, its development and implementation are beyond the scope of the present work and are therefore not described in detail. The resulting publication frequency measurements were subsequently used for downstream analyses.

The related publications and experiments were categorized into four sequential stages, i.e., in vitro, animal, clinical, and clinical trial, using BioBERT enhanced with active learning. We define in vitro as experiments using cell lines, animal studies as experiments using animal tissues or animals, clinical study as experiments using human tissues or human patients, and clinical trials are exclusively clinical trials. This allows us to quantify the research progression. Next, a straightforward approach was adopted to match publications with all known gene aliases, enabling precise attribution of research outputs to their corresponding genes. Finally, the data are reorganized in chronological order to capture the temporal evolution of gene-specific research. This method offers a transparent and scalable framework for evaluating research advances in the context of cancer therapeutics.

## 3. Results

**Figure 1:**
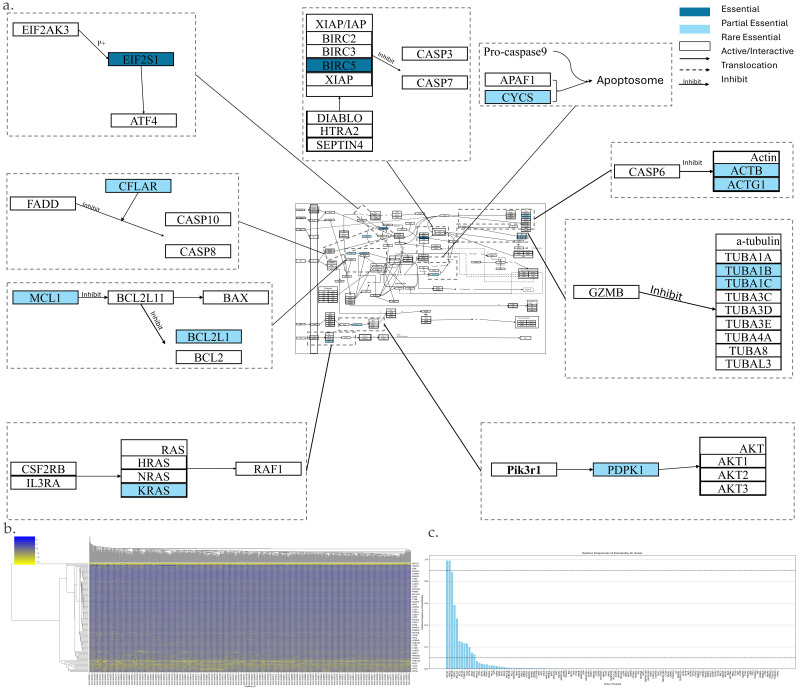
Essential gene analysis in the Apoptosis cell death pathway. (**a**) Apoptosis pathway with zoomed regions showing the essential or partial essential gene and its upstream and downstream interactions. (**b**) Heatmap of gene fitness effect score hierarchical clustering. (**c**) Bar plot showing the frequency of gene essentiality across cell lines.

The apoptosis pathway is shown in Figure 1a,b. It has 134 genes. Among them, there are 2 AEGs, 10 PEGs, and 122 REGs (Figure 1c). The following is an illustration of key AEGs and PEGs, which are underlined.

- EIF2S1 *(eIF2α)* promotes Apoptosis and is activated and phosphorylated in response to ER stress. This activation can lead to the activation of ATF4 and subsequent transcription of DDIT3 which can induce the Apoptosis [114,115,116].
- CFLAR (also known as c-FLIP) is a key anti-apoptotic protein. It competes with FADD to bind, thereby inhibiting its interaction with CASP8 (Caspase-8) and CASP10 (Caspase-10), which are critical components of inducing the extrinsic apoptotic pathway [117,118,119,120].
- MCL-1 is an anti-apoptotic protein that binds to BCL2L11, thereby playing an inhibitory role in regulating its pro-apoptotic activity [121,122,123]. BCL2L11 can bind to the anti-apoptotic protein BCL2L1, preventing it from inhibiting Apoptosis [124,125].
- *BIRC5*, a member of the inhibitor of Apoptosis (IAP) gene family, inhibits the function of Caspase-7 and negatively regulates Apoptosis. Its function can be inhibited by DIABLO/HTRA2/ARTS (also known as Septin4) [126,127,128].
- CYCS (Cytochrome c) is a pro-apoptotic protein which is released by the mitochondrial outer membrane permeabilization (MOMP). CYCS interacts with APAF1 to activate CCaspase-9, which is crucial for the activation of Caspase-3 [129,130,131,132].
- α-Tubulin is a substrate of GZMB. Its cleavage disrupts microtubule function, which is critical for maintaining cell shape and integrity [133,134,135].
- Actin, on the other hand, is a substrate of Caspase-6. The cleavage of actin contributes to cell shrinkage and further compromises the cellular structure [136,137,138].
- PDPK1 (PDK1) promotes cell survival. It is located on the inner side of the cell membrane. PDPK1 binds to PIP3 and recruits Akt simultaneously. The formation of this complex leads to the phosphorylation and activation of Akt by PDPK1 [139,140,141,142].
- KRAS, a subunit of the RAS protein, contributes to cell survival by inducing the expression of the pro-survival gene *BCL2* [143,144].

**Figure 2:**
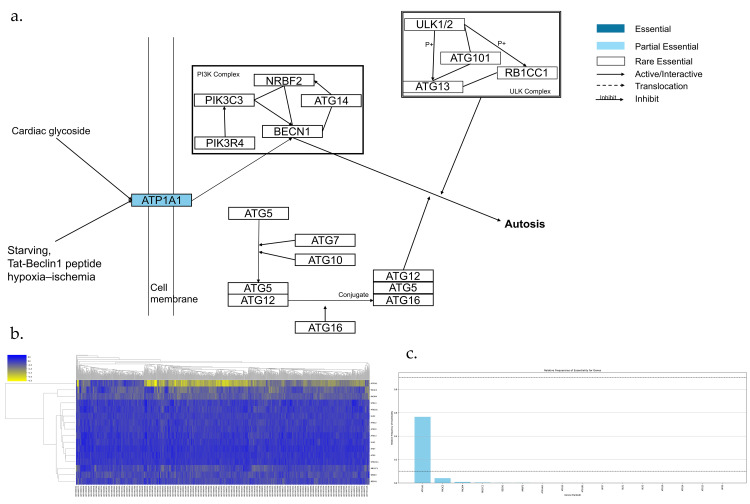
Essential gene analysis in the Autosis cell death pathway. (**a**) Autosis pathway with partial essential gene and its upstream and downstream interactions. (**b**) Heatmap of gene fitness effect score hierarchical clustering. (**c**) Bar plot showing the frequency of gene essentiality across cell lines.

Autosis pathway is shown in Figure 2a,b, which has 16 genes. Among them, there are no AEGs, 1 PEG, and 15 REGs (Figure 2c). ATP1A1 is a subunit of the Na^+^/K^+^-ATPase. It is a transmembrane protein which transports iron in and out of a cell. Although the downstream pathway remains unknown [8], there is solid evidence that Autosis is an Autophagy-dependent cell death pathway and Na^+^ and K^+^-ATPase work together to induce the Autosis [24,25,26,27].

**Figure 3:**
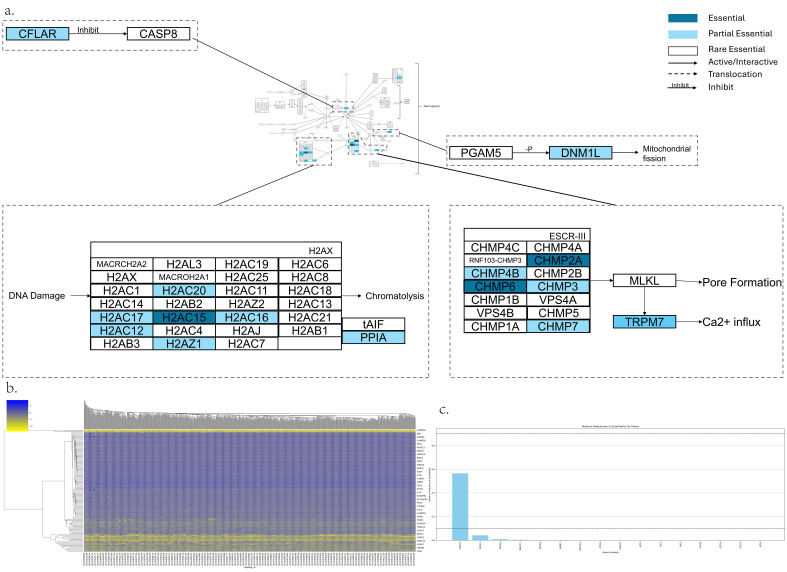
Essential gene analysis in the Necroptosis cell death pathway. (**a**) Necroptosis pathway with zoomed regions showing the essential or partial essential gene and its upstream and downstream interactions. (**b**) Heatmap of gene fitness effect score hierarchical clustering. (**c**) Bar plot showing the frequency of gene essentiality across cell lines.

The Necroptosis pathway is shown in Figure 3a,b, which has 148 genes. The following is a summary of key AEGs and PEGs. *CHMP2A, H2AC15,* and *CHMP6* were found to be AEGs in most tumor cells. Additionally, the following genes were identified as PEGs: *H2AC17, CHMP4B, H2AC16, H2AC12, TRPM7, CHMP3, CFLAR, DNM1L, H2AC20, VDAC1, PPIA, H2AZ1,* and *CHMP7*. The remaining genes were classified as REGs (Figure 3c).

- H2AC15, H2AC16, H2AC17, H2AC12, H2AC20 and H2AZ1 are a subunit of H2AX. It plays a critical role in DNA damage repair. It functions to recruit and accumulate DNA repair protein to the sites of double strand damage [145]. The DNA damage will induce the tAIF (also known as AIFM1) to move from mitochondria to the nucleus. Subsequently, tAIF binds to PPIA and H2AX and induces the chromatolysis which induces the necrosis [146,147].
- CHMP4B, CHMP2A, CHMP6, CHMP7 and CHMP3 are a component of ESCRT-III. ESCRT-III binds to activated MLKL, forms a plasma membrane, and results in Necroptosis [148,149].
- TRPM7 is involved in MLKL-mediated Necroptosis. It is an MLKL downstream target for the mediated Ca^2+^ influx and induces Necroptosis [149,150].
- CFLAR is an anti-Apoptosis protein which inhibits the activation of CASP8 (Caspase-8). CASP8 inactivation leads to RIPK1 interaction with RIPK3, and forms complex IIb, which promotes Necroptosis. After necrosis binds to MLKL, this complex binds to PGAM5L, and results in PGAM5L phosphorylation and activation. Subsequently, activated PGAM5L dephosphorylates and activates DNM1L (Drp1), a mitochondrial fission protein inducing a mitochondrial fragment which is critical to necrosis execution [151].
- *VDAC1* is described in the MPT cell death pathway.

**Figure 4:**
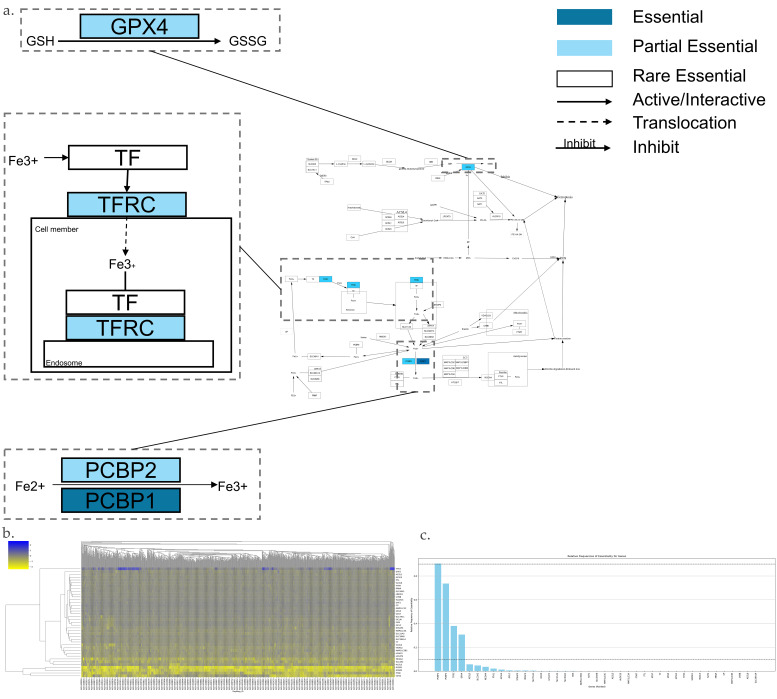
Essential gene analysis in the Ferroptosis cell death pathway. (**a**) Ferroptosis pathway with zoomed regions showing the essential or partial essential gene and its upstream and downstream interactions. (**b**) Heatmap of gene fitness effect score hierarchical clustering. (**c**) Bar plot showing the frequency of gene essentiality across cell lines.

The Ferroptosis pathway is shown in Figure 4a,b, which has 41 genes. Among them, there is 1 AEG, 3 PEGs, and 37 REGs (Figure 4c). The following is a summary of key AEGs and PEGs.

- GPX4 plays a central role in inhibiting Ferroptosis. GPX4 converts GSH to GSSH and reduces the ROC [152]. Accumulation of ROS could induce the Ferroptosis.
- TFR1, also known as TFRC, is a transferrin receptor on the cell membrane [153]. It binds with Fe^3+^. With the help of the TF receptor, which is a cell membrane protein, this complex is taken into the cell through endocytose. Taken into the cell, Fe^3+^ is reduced to Fe^2+^, and it is stored in a labile iron pool. This storage process helps prevent Ferroptosis [154,155].
- The Fe^2+^ is transported by divalent metal transporter 1, or zinc–iron regulatory protein family 8/14, to the cytoplasm [7]. Subsequently, PCBP1 and PCBP2, as a cytosolic iron chaperone, deliver iron to ferritin, forming a non-toxic storage complex [156,157].

**Figure 5:**
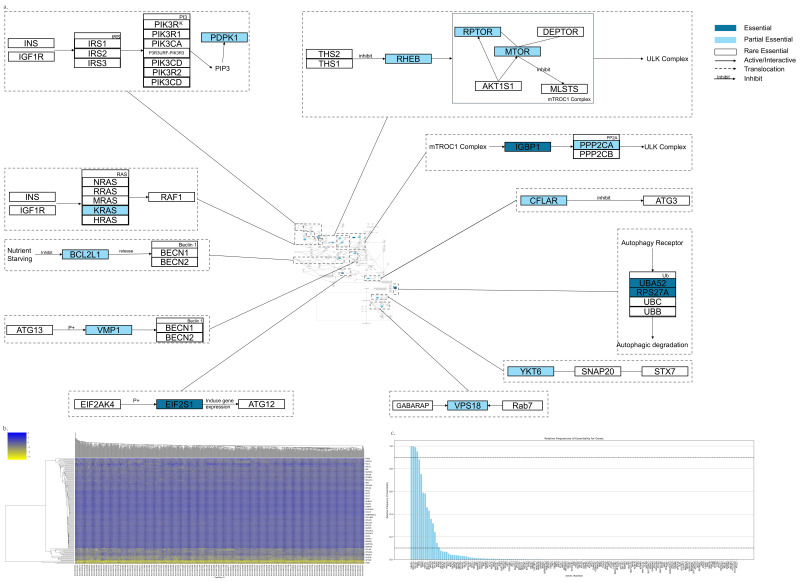
Essential gene analysis in the Autophagy cell death pathway. (**a**) Autophagy pathway with zoomed regions showing the essential or partial essential gene and its upstream and downstream interactions. (**b**) Heatmap of gene fitness effect score hierarchical clustering. (**c**) Bar plot showing the frequency of gene essentiality across cell lines.

The Autophagy pathway is shown in Figure 5a,b, which has 167 genes. Among them, there are 4 AEGs, 11 PEGs, and 152 REGs (Figure 5c). The following is a highlight of key AEGs and PEGs.

- The outside singling activation (from IRS or IGF1R) leads to the IRS phosphorylate. Then phosphorylated IRC interacts with PI3K and produces PIP3, and PIP3 recruits PDPK1 and AKT [158]. AKT can phosphorylate and inactivate the TSC1/2 complex, which is an inducer of Autophagy [159].
- KRAS is a component of RAS. INS or IGFR binding to RAS leads to the activation of MAPK signaling, which then inhibits the Autophagy [160].
- RPTOR functions as an adaptor for some mTORC1 substrates, and it targets the mTORC1 into the Lysosome [161]. Raptor and mTOR are compounds of the mTROC1 complex which play a central role in Autophagy [162]. mTORC1 is a repressor of the Autophagy thought interacting with ULK complex. When Rheb binds to GTP, it activates the mTORC1 complex through directly binding to mTOR [163]. This process can be inhibited by the complex formed by Tsc1 and Tsc2 [164,165].
- PPP2CA is a component of PP2A which regulates Autophagy independent of mTORC1 through dephosphorylating the ULK complex [166]. PPP2CA is critical to inducing the Autophagy. IGBP1, also known as alpha4, binds to PP2A and inhibits PP2A’s dephosphorylate activity. mTOR can regulate the formation of the IGBP1-PP2A complex [167,168].
- BCL2Ll binds and inhibits Beclin-1, which is critical to mediating Autophagy during non-starvation conditions and inhibits its Autophagy function. The starvation leads to the phosphorylation of BCL2L1, and leads to its disassociation from Beclin-1 [169].
- VMP1 binds to Beclin1 and contributes to the formation of the PI3K complex, a positive regulator of Autophagy [170].
- EIF2S1 phosphorylation is important in inducing Autophagy. It can be phosphorylated by EIF2AK4 and EIF2AK3 [171]. Phosphorylated EIF2S1 can promote the expression of ATG12 [172].
- PRS27A and UBA52 are ubiquitin proteins. Ubiquitin protein is tag for autophagic degradation which could be recognized by Autophagy receptors [173,174].
- YKT6 is located on ER. It belongs to the SNARE complex. The SNARE complex mediates the fusion between autophagosomes and Lysosomes, which is regulated by HOPS (the core subunit is VPS18). HOPS is recruited to the membrane by binding to RAB7 on Lysosomes. On the autophagosome side, the HOPS complex binds to STX17 and LC3 proteins [175].
- CFLAR is an Autophagy inhibitor which inhibits the function of ATG3 [118,176].

**Figure 6:**
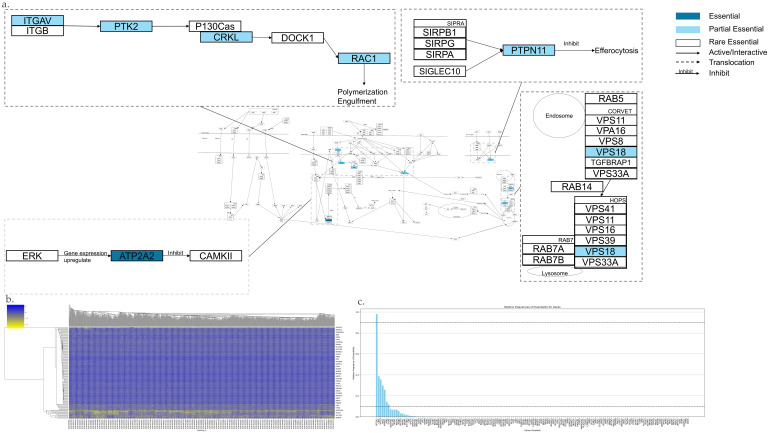
Essential gene analysis in the Efferocytosis cell death pathway. (**a**) Efferocytosis pathway with zoomed regions showing the essential or partial essential gene and its upstream and downstream interactions. (**b**) Heatmap of gene fitness effect score hierarchical clustering. (**c**) Bar plot showing the frequency of gene essentiality across cell lines.

The Efferocytosis pathway is shown in Figure 6a,b, which has 156 genes. Among them, there is 1 AEG, 6 PEGs, and 149 REGs (Figure 6c). The following is a highlight of key AEGs and PEGs.

- Ligand binds to receptor integrin α3/α5 (ITGAV and ITGB). Subsequently, FAK (also known as PTK2) is recruited by integrin α3/α5. It leads to the phosphorylation of P130CAS and recruitment of p130CAS. *CRKL* encodes the CRK-like protein. The CRK-DOCK180 (also known as DOCK1) complex is recruited by the phosphorylation of P130Cas. Ultimately, it activates RAC1, which plays a central role in the Efferocytosis process [177,178,179,180].
- *ATP2A2* encodes one of the SERCA2 proteins. The “eat me” signal binds to its receptor, leading to the activation of ERK. This activation results in the ERK-mediated expression of the *ATP2A2* gene. This process decreases cytosolic Ca^2+^ concentration and suppresses CaMKII activity [181].
- During early endosomal fusion events, CORVET binds to Rab5 and functions as a general tether to promote endosomal fusion. When early phagosomes transition to late phagosomes, Rab5 is replaced by Rab7. It mediates the fusion of late endosomes with Lysosomes. At the late endosome stage, Rab7 interacts with the HOPS complex. It facilitates the tethering of late endosomes to vacuoles [182].
- Receptor signal-regulatory protein-α (SIRPα) and SIGLEC10 on macrophages can inhibit engulfment through SHP1 (also known as PTPN11) and inhibit the Efferocytosis. SIRPα phosphorylation enables the docking and recruitment of SHP-2. The inhibition of Efferocytosis is mediated by the “do not eat me” mechanism. This process is primarily mediated by CD47, which binds to SIRPα. CD47 has been reported to be overexpressed on the cell membrane of many human cancer cells, allowing tumor cells to evade phagocytosis [183,184].

**Figure 7:**
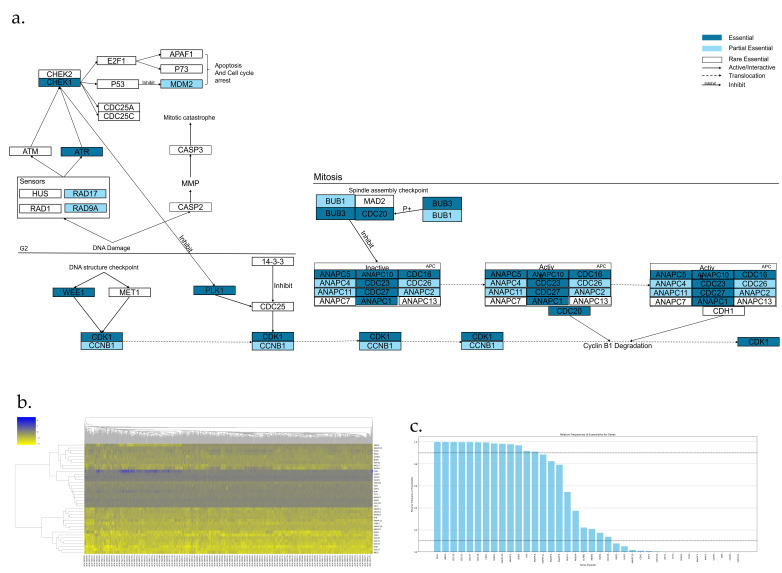
Essential gene analysis in the Mitotic mediated cell death pathway. (**a**) Mitotic cell death pathway with essential or partial essential gene and its upstream and downstream interactions. (**b**) Heatmap of gene fitness effect score hierarchical clustering. (**c**) Bar plot showing the frequency of gene essentiality across cell lines.

The Mitotic mediated cell death pathway is shown in Figure 7a,b, which has 38 genes. Among them, there are 13 AEGs, 9 PEGs, and 16 REGs (Figure 7c). The following is a highlight of key AEGs and PEGs.

- “DNA structure checkpoints” activate Wee1, which in turn inactivates CDK1 through phosphorylation [30].
- PLK1 serves as an endogenous repressor of Mitotic catastrophe. During the G2 phase of mitosis, Chk1 inhibits PLK1, which is necessary for the activation of CDC25. The inhibition of PLK1 results in spindle defects, and ultimately leads to Apoptosis [185,186].
- BUB3 and Cdc20 are components of the spindle assembly checkpoint, which functions to arrest the activation of the APC complex. The assembly of the spindle checkpoint begins with BUB3 binding to BUB1, followed by the recruitment of CDC20 by the BUB1-BUB3 complex [187]. Subsequently, MAD2 is recruited to the BUB1-BUB3-CDC20 complex, which functions to bind and inhibit the activation of the APC/C-Cdc20 protein [188]. The APC complex includes subunits CDC27, CDC26, CDC23, CDC16, ANAPC10, ANAPC5, ANAPC4, ANAPC2, ANAPC1 and ANAPC11. APC is active during anaphase and is responsible for the degradation of cyclin B1 [189].
- The CDK1/CCNB1 (also known as Cyclin B1) complex is essential for mitosis and Mitotic catastrophe. During the G2 phase, “DNA structure checkpoints” stimulate Wee1 and Myt1, which will inactivate CDK1 [30,31,190].
- Mitotic catastrophe can be induced by aberrant Mitotic entry, caused by premature activation of the CDK1/Cyclin B1 complex or prolonged inhibition of APC. DNA damage in the G2 phase can be detected by RAD17 and RAD9, which activates ATR, leading to the activation of Chk1. Chk1 phosphorylates p53, stabilizing it and leading to the loss of MDM2’s p53 degradation function. Consequently, the cell eventually undergoes Apoptosis [9,36,37].

**Figure 8:**
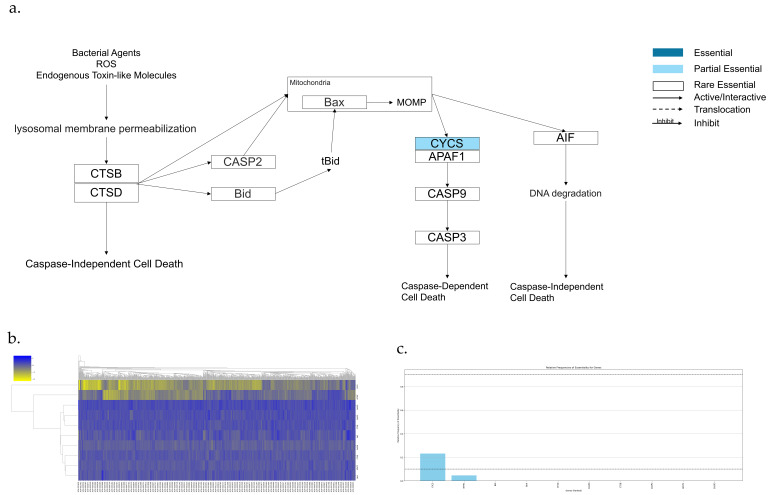
Essential gene analysis in the Lysosome-dependent cell death pathway. (**a**) Lysosome-dependent cell death pathway with partial essential gene and its upstream and downstream interactions. (**b**) Heatmap of gene fitness effect score hierarchical clustering. (**c**) Bar plot showing the frequency of gene essentiality across cell lines.

The Lysosome-dependent cell death pathway is shown in Figure 8a,b, which has 10 genes. Among them, there are no AEGs, 1 PEG, and 9 REGs (Figure 8c). Cytochrome c (Also known as CYCS) is a Lysosome-inducing gene. MOMP causes the release of cytochrome c into the cytosol. Then, cytochrome c interacts with apoptotic protease-activating factor (Apaf-1) to activate Caspase-9 (also known as CASP9) [40,47,191].

**Figure 9:**
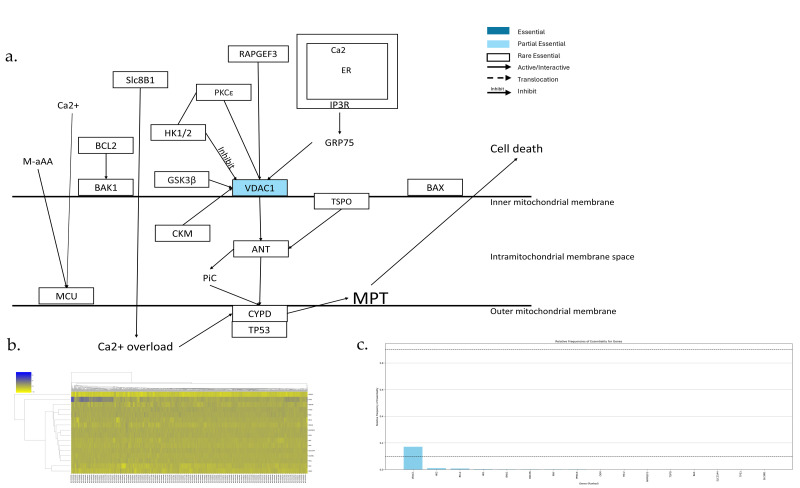
Essential gene analysis in the MPT cell death pathway. (**a**) MPT cell death pathway with partial essential gene and its upstream and downstream interactions. (**b**) Heatmap of gene fitness effect scores hierarchical clustering. (**c**) Bar plot showing the frequency of gene essentiality across cell lines.

The MPT-driven necrosis pathway is shown in Figure 9a,b, which has 16 genes. Among them, there are no AEGs, 1 PEG, and 15 REGs (Figure 9c). VDAC1 is a component of PTPC, which is the core protein of MPT-drive necrosis. There are several mechanisms which could regulate VDAC1, which either induce or inhibit MPT. Active GSK3b could phosphorylate VDACs and induce MPT [58]. PKCɛ can stabilize the HK2 binding to VDAC1 and inhibit MPT [59]. BCL-2 can inhibit MPT through regulating VDAC1 [60,61]. BAX and BAK could induce MPT through interacting with ANT and VDAC1 [62,63,64]. BAD could trigger MPT through a VDAC1-dependent BCL-xl responsive mechanism [65]. However, there are many studies that showed that the inactivation of VDAC1 and ANT is dispensable for MPT regulation and execution [52,53,54,55].

**Figure 10:**
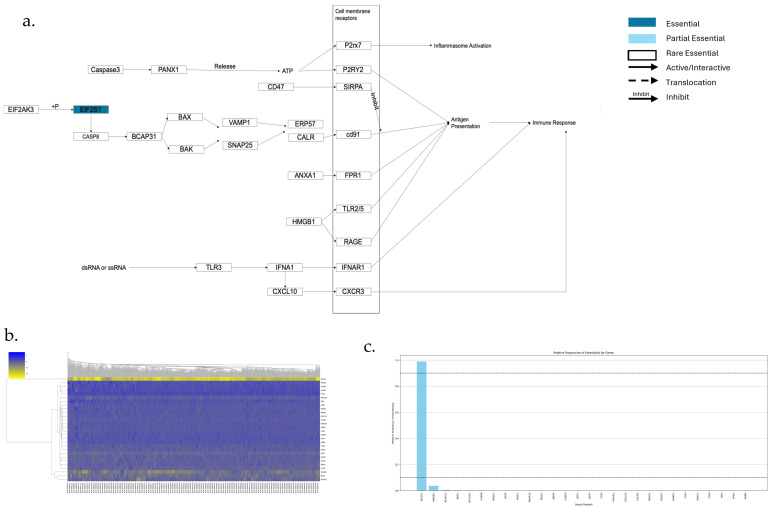
Essential gene analysis in the Immunogenic cell death pathway. (**a**) Immunogenic cell death pathway with partial essential gene and its upstream and downstream interactions. (**b**) Heatmap of gene fitness effect scores hierarchical clustering. (**c**) Bar plot showing the frequency of gene essentiality across cell lines.

The Immunogenic cell death pathway is shown in Figure 10a,b, which has 28 genes. Among them, there is 1 AEG, no PEGs, and 27 REGs (Figure 10c). CALR translocation occurs at the early stage of Immunogenic cell death. The Phosphorylation of EIF2S1 by EIF2AK3 activates Caspase-8, which then cleaves BAP31 to regulate CALR translocation.

Parthanatos and Pyroptosis have no AEGs or PEGs. Their pathways and gene essentiality data are shown in Supplementary Figures S1 and S2.

**Figure 11:**
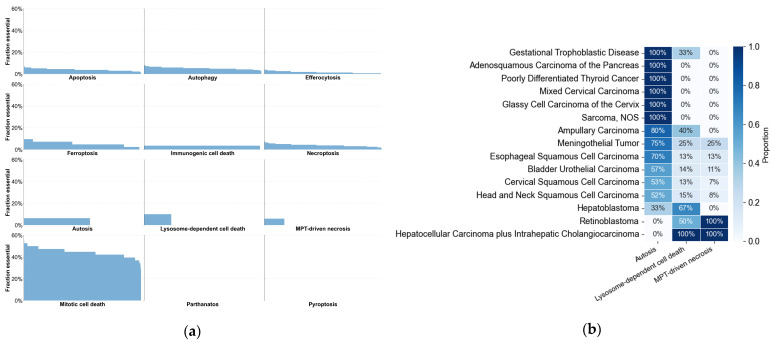
Gene essentiality fraction across cell death pathways and cancer type enrichment of three selected pathways. (**a**) Fraction of essential genes per pathway across cancer cell lines. (**b**) Cancer type proportion among cell lines with essential fraction in three selected pathways: Autosis, Lysosome-dependent cell death, and MPT-driven necrosis.

To further assess pathway-level essentiality patterns based on AEGs and PEGs, we performed additional analyses across cell death pathways and cancer types. For each cell line, the pathway-level proportion was calculated as the number of AEGs and PEGs identified as essential in that cell line divided by the total number of genes annotated to that pathway, including AEGs, PEGs, and REGs. We observed that, for most cell death pathways, the pathway essentiality fraction was relatively consistent across cancer cell lines, suggesting broadly similar pathway relevance across cancer contexts. In contrast, three pathways (Autosis, Lysosome-dependent cell death, and MPT-driven necrosis) displayed clear separation in pathway essentiality fractions (Figure 11a). These results suggest that these pathways may have greater functional importance in some cell lines than others. We therefore further analyzed the cancer type distribution of these selected cell lines. Cancer type fraction was calculated as the number of selected cell lines in each cancer type divided by the total number of cell lines belonging to that cancer type.

Cancer type enrichment analysis, i.e., >50% enrichment, revealed distinct patterns across the three selected cell death pathways. Autosis appeared to be enriched across a broader range of cancer types (n = 12). In contrast, Lysosome-dependent cell death (n = 3) and MPT-driven necrosis (n = 2) were enriched in fewer cancer types. Notably, both pathways showed high enrichment in hepatocellular carcinoma plus intrahepatic cholangiocarcinoma (cHCC-CCA), a rare and aggressive primary liver cancer [192], and in retinoblastoma, an eye cancer. Lysosome-dependent cell death also showed high enrichment in hepatoblastoma, a childhood liver cancer (Figure 11b). These findings suggest that targeting these cell death pathways may have potential therapeutic relevance in specific cancer types.

**Figure 12:**
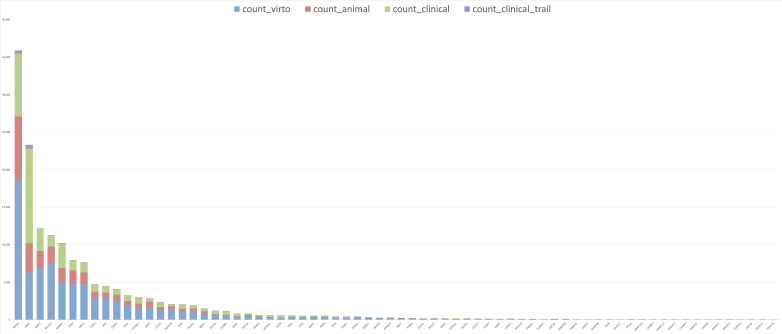
Stacked bar chart showing the number of gene-related publications across different study categories (in vitro, animal, clinical study, and clinical trial).

To evaluate the potential of the identified genes in clinical application, we retrieved the frequency of publications for each AEG and PEG from PubMed and categorized them into four groups based on the model system used (Figure 12). Our analysis revealed that only a few genes have a high number of publications. *MTOR* has the highest count, with 35,899 publications, followed by *KRAS* with 23,329. Additionally, three genes (*BIRC5*, *BCL2L1*, and *MDM2*) have between 10,000 and 20,000 publications, while 15 genes fall within the range of 1000 to 10,000 publications. In contrast, 25 genes have fewer than 100 publications, with *H2AC16* being almost unstudied, having only two publications in PubMed. When analyzing the distribution of publications across different study models, we found that most research is still conducted in in vitro systems, with a total of 80,091 publications. In contrast, clinical studies are significantly underrepresented, with only 1379 publications reported.

## 4. Discussion

The cell death pathway is a fundamental biological process. For many years, people believed cell death pathways were conserved as running in parallel and have no overlap. However, based on the years of publications, researchers realized that they are overlapping and could interact with each other through the interconnected, or even overlapping, signaling pathways. This has also been validated by our data; during the collection of genes, we noticed that there are 119 genes which were found to function in more than one cell death pathway.

Cell death pathways are essential for organismal development and the maintenance of tissue homeostasis [193]. However, when these pathways are dysregulated, they can contribute to the development and progression of disease. In cancer, impaired activation of cell death pathways allows tumor cells to evade cell death and promotes tumor progression. In addition, cell death pathways are also involved in autoimmune and inflammatory diseases [194]. For example, neutrophil Ferroptosis has been reported to contribute to neutropenia in systemic lupus erythematosus, and the deletion of genes such as RIPK3, Caspase-1, and Caspase-8 has been shown to reduce inflammatory disease progression in mice, which may be related to the crosstalk among Pyroptosis, Apoptosis, and Necroptosis. The expression of BCL2 family genes is also closely associated with breast cancer development and progression [195,196]. At the same time, the clinical importance of cell death pathways has been demonstrated in cancer therapy, as several BCL2-targeting drugs have been approved by the FDA and have shown remarkable efficacy with manageable toxicities in hematologic malignancies through the induction of Apoptosis [197]. Together, these findings indicate that cell death pathways are closely associated with disease pathogenesis as well as therapeutic intervention. Therefore, further investigation of cell death-related genes may provide important insight into the identification of novel cancer therapeutic targets, highlighting the significance of our study.

Overall, among these 12 cell death pathways, 23, 47, and 549 genes were classified as always essential, partial essential, and rare essential, respectively. In two cell death pathways, Parthanatos, and Pyroptosis, all genes were rare essential. Among the other ten cell death pathways, Apoptosis, Autosis, Necroptosis, Efferocytosis, Ferroptosis, Mitotic cell death, Autophagy, Lysosome cell death, MPT-driven necrosis and Immunogenic, there are (10, 1, 13, 6, 3, 9, 11, 1, 1, 0) partial essential genes, and (2, 0, 3, 1, 1, 13, 4, 0, 0, 1) always essential genes. These cell death pathway essential genes could be viable targets for therapeutic drug development for cancer therapies.

Although most cell death pathways showed similar essentiality patterns across cancer cell lines, Autosis, Lysosome-dependent cell death, and MPT-driven necrosis exhibited more distinct patterns. This suggests that these three pathways may have more context-dependent relevance in specific cancer types. This interpretation was further supported by the cancer type enrichment analysis. Together, these findings provide a rationale for further biological validation and suggest that these pathways may be explored in future functional and therapeutic studies.

The data publication count highlights the potential of the identified genes as cancer therapy targets, as many of them remain largely unstudied. Additionally, for genes with a high number of publications, we believe they may provide meaningful insights, given the well-established in vitro research but limited clinical trials. In further study, by using the CRISPR-Cas9 system, we could further explore their potential in clinical cancer therapy.

Genome-wide CRISPR-Cas9 technology is a powerful tool to screen essential genes in cancer cells. Although many genes are found partial or always essential in nine out of twelve cell death pathways, these data shall be interpreted cautiously. Among always essential genes, they are remained to be further validated through both on-target and off-target experiments [198,199], because of potential off-target issues in CRISPR-cas9 [200,201]. Among partial essential genes, besides the same on-target and off-target issues, there is another interesting question on why a gene is essential in some cancer cell, but not essential in the others? While there is research in the literature that attempts to explain and predict gene essentiality using genomics, transcriptomics and signaling pathway data [202,203], they are not yet studied among essential genes in the cell death pathways.

In this paper, twelve cell death pathways have covered 600 relevant genes among 18,443 genes that were screened in the DepMap. Other than those 600, there are hundreds of genes, which are either always essential or partial essential among 1150 cancer cells. However, their roles or relationships with those cell death pathways remain to be investigated.

Gene essentiality itself only explains its importance in cell viability and its role in a certain cell death pathway. It remains unclear whether and how two or more cell death pathways depend on each other in regulating the cell death. Even within a cell death pathway, it is also interesting to know whether two genes or multiple genes have redundancy roles, or their functions depend on each other. For example, BRAC1 and PARP1 control two parallel routes in the DNA damage pathway. They have a synthetic lethal relationship. These interesting questions shall rely on experiments and data from CRISPR-Cas9 gene combination double knockout technology [204].

## Data Availability

The gene essentiality data were obtained from the Dependency Map (DepMap) portal (https://depmap.org/portal/) (accessed on 11 November 2024). Specifically, the dataset is available via the following link: https://plus.figshare.com/articles/dataset/DepMap_24Q2_Public/25880521/1 (accessed on 11 November 2024). This dataset provides a gene essentiality score for 18,443 genes among 1150 cancer cell lines [12].

## Supplementary Materials

The following supporting information can be downloaded at: https://www.mdpi.com/article/10.3390/genes17040491/s1, Figure S1: Essential gene analysis in the Parthanatos cell death pathway. (a) Parthanatos cell death pathway. (b) Heatmap of gene fitness effect scores hierarchical clustering. (c) Bar plot showing the frequency of gene essentiality across cell lines; Figure S2: Essential gene analysis in the Pyroptosis cell death pathway. (a) Pyroptosis cell death pathway. (b) Heatmap of gene fitness effect scores hierarchical clustering. (c) Bar plot showing the frequency of gene essentiality across cell lines.

## Abbreviations

The following abbreviations are used in this manuscript:

NCCD	Nomenclature Committee on Cell Death
ROS	Reactive Oxygen Species
MPT	Mitochondria Permeability Transition
AEG	Always essential genes
REG	Rare essential genes
PEG	Partially essential genes
CASP8	Caspase-8
CASP10	Caspase-10
IAP	Inhibitor of Apoptosis
MOMP	The mitochondrial outer membrane permeabilization
