# Supplementary material for: Landscape of Gene Essentiality in Cancer Cell Death Pathways
Source: Genes (Basel). 2026 Apr 21;17(4):491. doi: 10.3390/genes17040491 (PMC13116478; doi:10.3390/genes17040491)
Supplement: Supplementary file 1 [file genes-17-00491-s001.zip › genes-4219992-supplementary.pptx]

## Slide 1
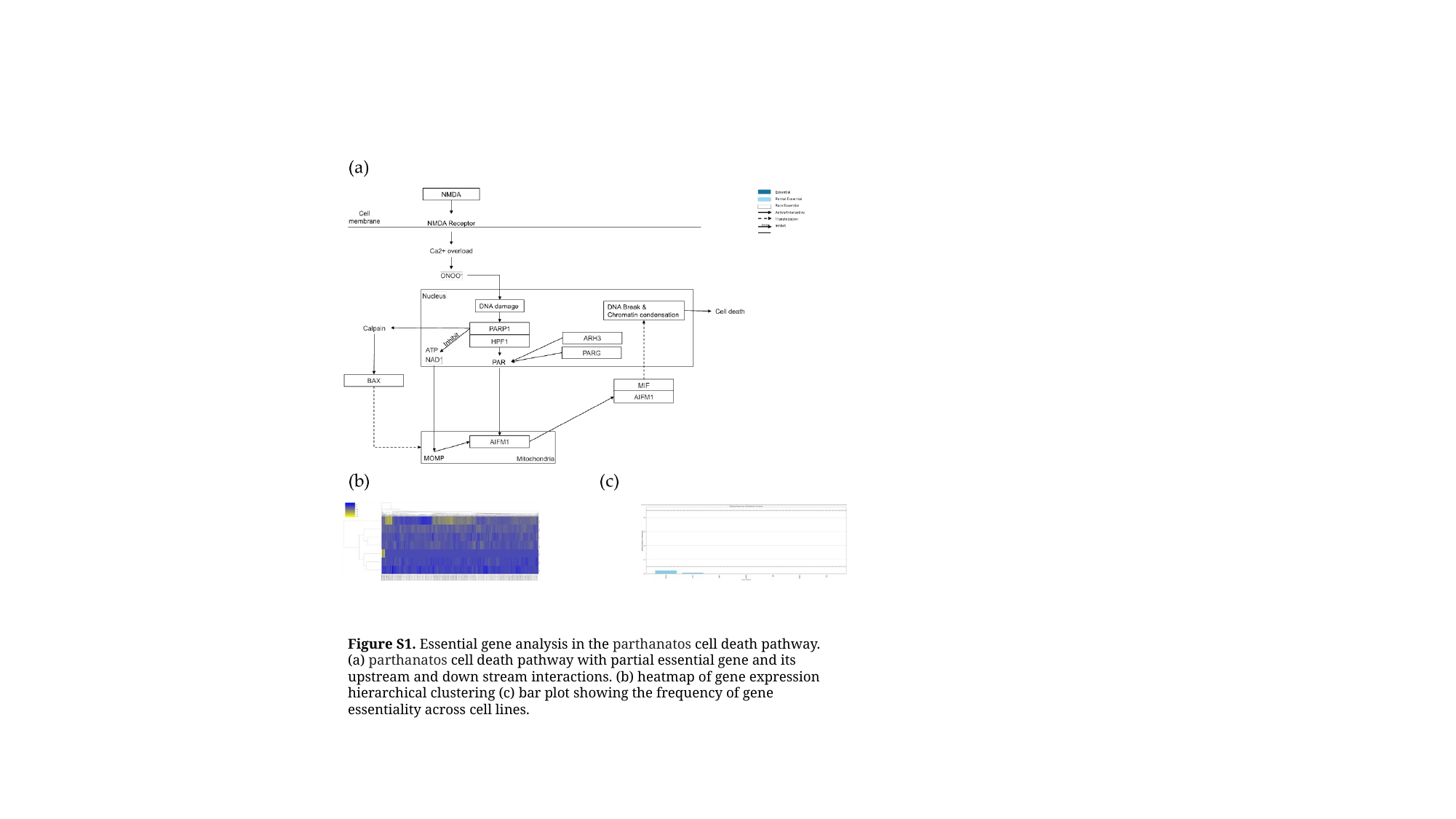

Figure S1. Essential gene analysis in the parthanatos cell death pathway. (a) parthanatos cell death pathway with partial essential gene and its upstream and down stream interactions. (b) heatmap of gene expression hierarchical clustering (c) bar plot showing the frequency of gene essentiality across cell lines.

## Slide 2
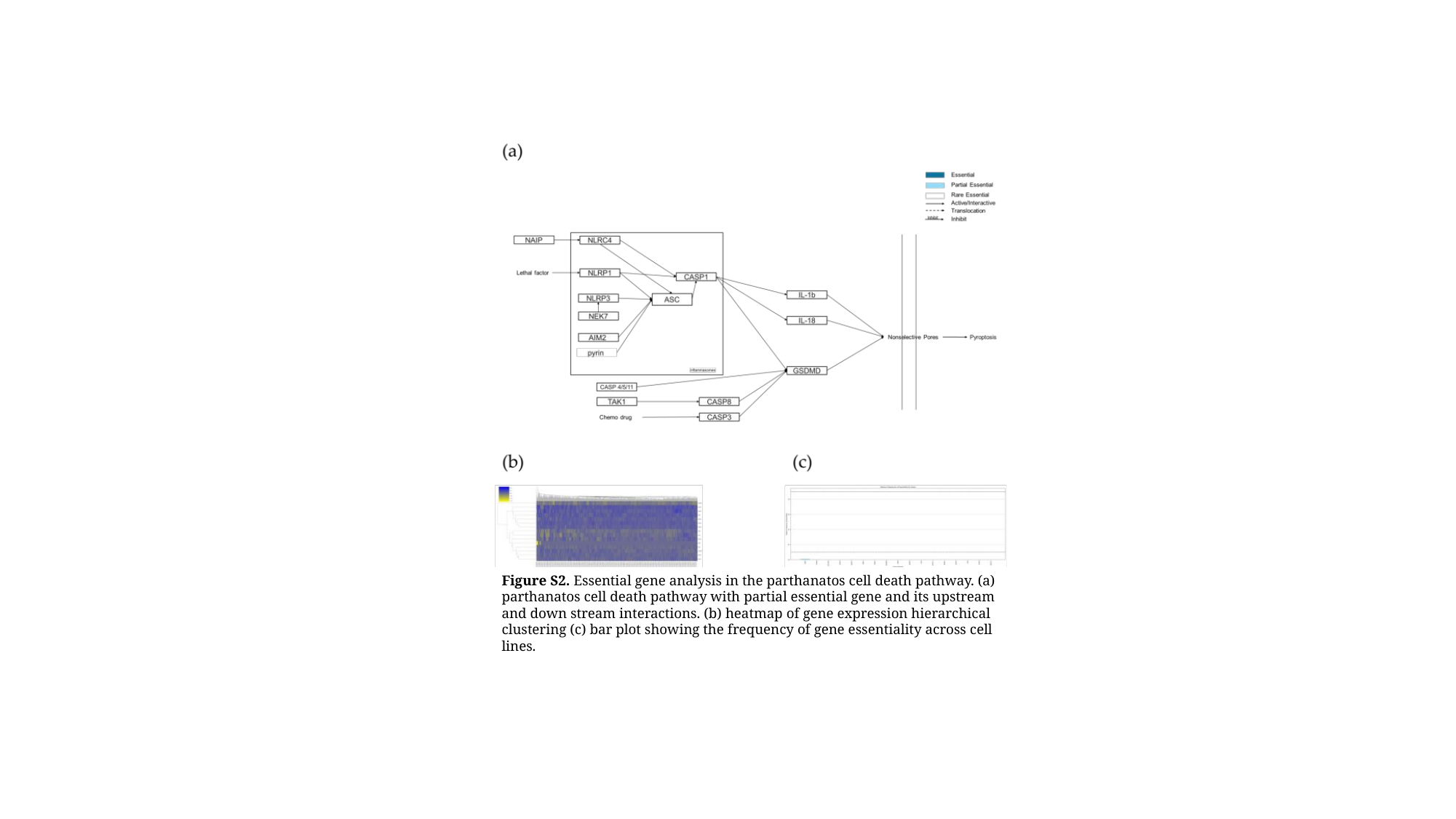

Figure S2. Essential gene analysis in the parthanatos cell death pathway. (a) parthanatos cell death pathway with partial essential gene and its upstream and down stream interactions. (b) heatmap of gene expression hierarchical clustering (c) bar plot showing the frequency of gene essentiality across cell lines.
